# Immune-evasive stem cells: engineering tolerance and reprogramming microenvironments for regenerative therapy

**DOI:** 10.1186/s13287-026-04997-4

**Published:** 2026-04-05

**Authors:** Xing Wu, Siyu Jin, Yufei Pan, Wenyu Zhen, Sensen Yu, Yulong Zhang, Fei Xu, Rui Wang, Mingyue Wu, Wansu Sun, Jianguang Xu, Xiaodong Zang, Hengguo Zhang

**Affiliations:** 1 College & Hospital of Stomatology, Anhui Provincial Key Laboratory of Oral Diseases Research, Anhui Medical University, Hefei, 230032 China; 2 Department of Biomedical Engineering, Institute for Quantitative Health Science and Engineering, Michigan State University, East Lansing, 48823 MI USA; 3 Department of pediatrics, Division of Life Sciences and Medicine, The First Affiliated Hospital of USTC, University of Science and Technology of China, Hefei, 230001 China

**Keywords:** Stem cell transplantation, Immune evasion immunomodulation, Clinical application

## Abstract

Stem cell transplantation (SCT) holds significant promise for regenerative medicine, yet immune rejection remains a major obstacle. To address this, recent advances leverage CRISPR/Cas9 to engineer hypoimmunogenic induced pluripotent stem cells. These modified cells lack classical immune recognition markers (HLA class I/II) yet retain immune-tolerant molecules such as HLA-E, HLA-G, and CD47, enabling their universal use across different individuals. Additionally, mesenchymal stem cell-derived exosomes and immune checkpoint modulators (e.g., PD-L1) have shown clinical effectiveness by reducing graft-versus-host disease and autoimmune reactions. They achieve this through mechanisms such as suppressing inflammatory T-cell activation, promoting regulatory T-cell expansion, and modulating macrophage polarization. Despite these advances, several challenges remain. One key concern is the potential tumorigenic risk caused by genomic instability during genome editing and long-term cell expansion. Emerging precision editing platforms, including base editing and prime editing, provide strategies to reduce double-strand DNA break–induced chromosomal rearrangements and improve genomic safety. Future research priorities include integrating AI-based immune profiling, precision genome editing, and advanced 3D-bioprinting technologies. Together, these innovations represent a paradigm shift toward developing safer, more effective, universally compatible stem cell therapies for diseases previously deemed untreatable.

## Introduction

As a cornerstone of regenerative medicine, stem cell transplantation (SCT) is a groundbreaking therapeutic strategy that restores or replaces damaged tissue. SCT has revolutionized the treatment landscape for various conditions, including hematologic malignancies, immune disorders, and genetic diseases [1–3]. Its reparative potential has opened new horizons for addressing previously intractable diseases.

SCT is broadly categorized into autologous, allogeneic, and syngeneic transplantation, each presenting distinct advantages and clinical challenges. Autologous SCT uses the patient’s own stem cells, significantly reducing immune rejection risk but potentially reintroducing malignant or defective cells. Allogeneic SCT provides broader therapeutic potential when autologous cells are unsuitable; however, it faces hurdles like immune rejection and graft-versus-host disease (GVHD). Syngeneic SCT involves genetically identical twins—virtually eliminating immune rejection—but remains clinically rare [4, 5]. Among these, allogeneic transplantation is especially critical for treating severe conditions, although immune compatibility remains a central challenge for optimizing graft survival and efficacy [6].

Recent clinical and translational advances underline the urgency of developing immune-evasive SCT strategies. As of December 2024, 116 interventional clinical trials involving pluripotent stem cell (hPSC)–derived products are underway globally, testing 83 unique products across conditions such as ocular, neurological, and oncologic diseases, without pervasive safety concerns [7–9]. Additionally, early-phase trials of neural stem cell transplantation for chronic ischemic stroke report clinically meaningful motor improvements at 12 months (Stanford University Clinical Trial, NCT04678462, 2024). showing an average Fugl-Meyer increase of 11.8 points. In orthopedics, a completed Phase III trial at Osaka University used arthroscopic transplantation of synovium-derived mesenchymal stem cell (MSCs) for knee cartilage defects (Osaka University, UMIN000014121, 2024). achieving stable cartilage repair over five years; results are currently under analysis.

This review systematically explores how stem cells evade or modulate immune responses to enhance engraftment and reduce rejection. It highlights recent breakthroughs in genetic engineering, exosome-based therapies, and immune checkpoint modulation. Moreover, it discusses clinical applications and proposes future directions to optimize immune escape and regulation. With an expanding clinical trial landscape, emerging neural and orthopedic successes, and unprecedented engagement in pluripotent stem cell translation, this review is both timely and essential to guide the next generation of universally compatible and clinically effective SCT strategies.

## Immune regulatory mechanisms in stem cell transplantation

In allogeneic stem cell transplantation (SCT), recipient immune cells often recognize donor stem cells as foreign. This response is primarily driven by human leukocyte antigen (HLA) molecules, which present peptides to T cells. Even minor HLA mismatches can activate T cells, induce antibody production, and recruit natural killer (NK) cells and macrophages. These immune responses frequently result in graft rejection and post-transplant complications [10–12].

Immune recognition involves both direct and indirect antigen presentation pathways. Donor antigens can be recognized directly by recipient T cells or indirectly through recipient antigen-presenting cells. These processes contribute to both acute and chronic rejection and strongly affect transplantation outcomes [13, 14]. Clinical and preclinical studies show that early immune sensitization compromises graft persistence. For example, donor-specific antibodies have been detected in a subset of allogeneic mesenchymal stem cell (MSC) recipients and are associated with reduced therapeutic efficacy [15]. Similarly, major histocompatibility complex (MHC)-mismatched induced pluripotent stem cell (iPSC)-derived grafts are rapidly rejected unless immune regulation is introduced [16].

**Fig. 1:**
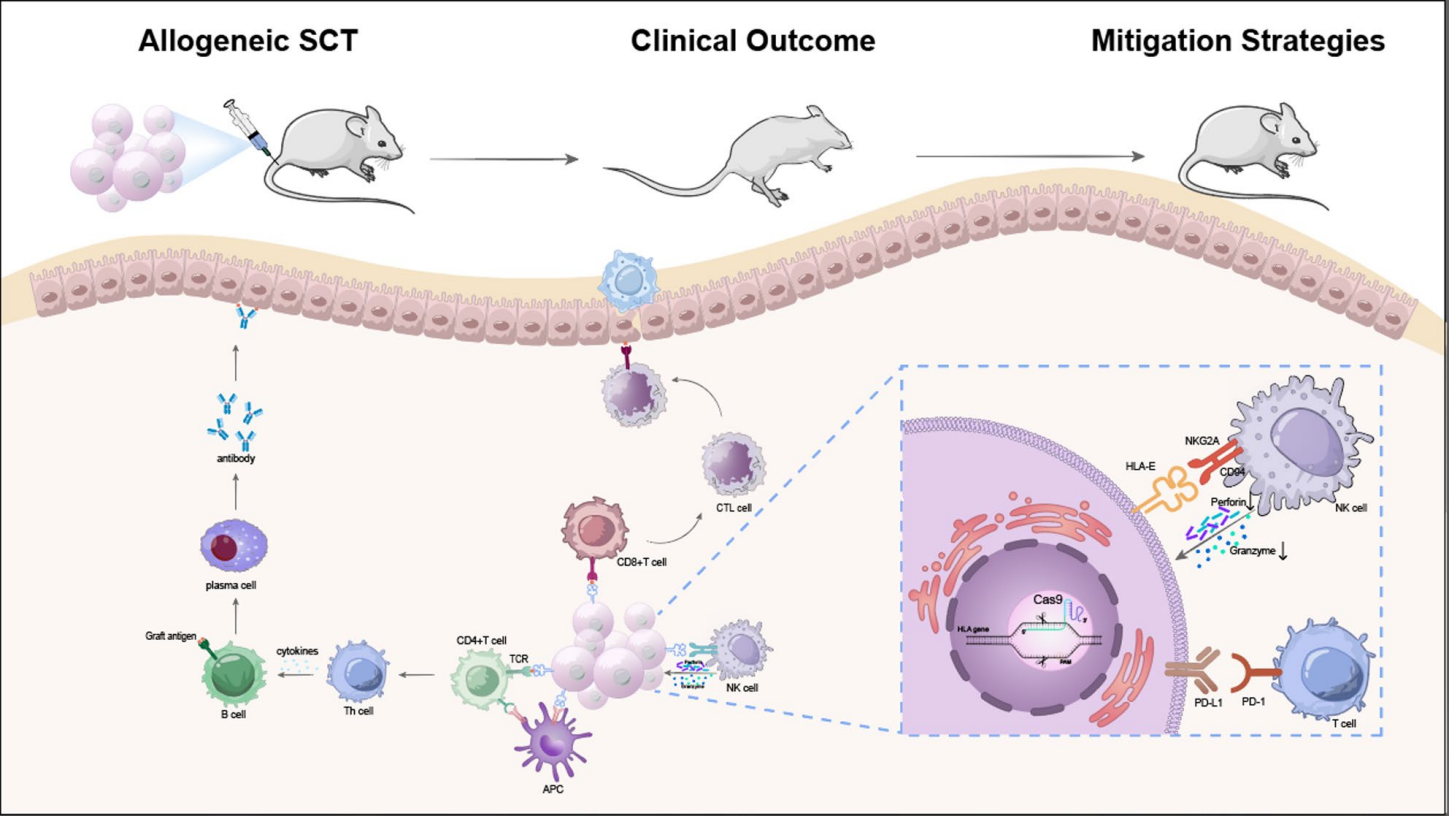
Immune response and mitigation strategies in allogeneic stem cell transplantation

These observations establish immune rejection as a central challenge in SCT and highlight the need to understand how stem cells interact with the host immune system. Importantly, stem cells are not passive immune targets. Instead, they actively shape immune responses through intrinsic immune-evasive properties and extrinsic regulatory mechanisms [17]. This chapter summarizes the core immune regulatory strategies employed by stem cells, providing a mechanistic framework for the translational and clinical applications discussed in subsequent chapters (Fig. 1).

After allogeneic stem cell transplantation (SCT), donor-derived cells are recognized by host antigen-presenting cells (APCs), which activate CD4 + T cells and CD8 + T cells, leading to cytotoxic T lymphocyte (CTL) responses and antibody production through B cell activation. Natural killer (NK) cells further contribute to graft rejection via perforin and granzyme release. These immune responses result in poor engraftment and clinical complications. To overcome these barriers, genome editing approaches such as CRISPR/Cas9 are applied to modify HLA expression, upregulate inhibitory ligands (e.g., HLA-E, PD-L1), and reduce T cell and NK cell-mediated cytotoxicity, thereby improving graft survival.

### Immune escape and regulatory functions of stem cells

**Fig. 2:**
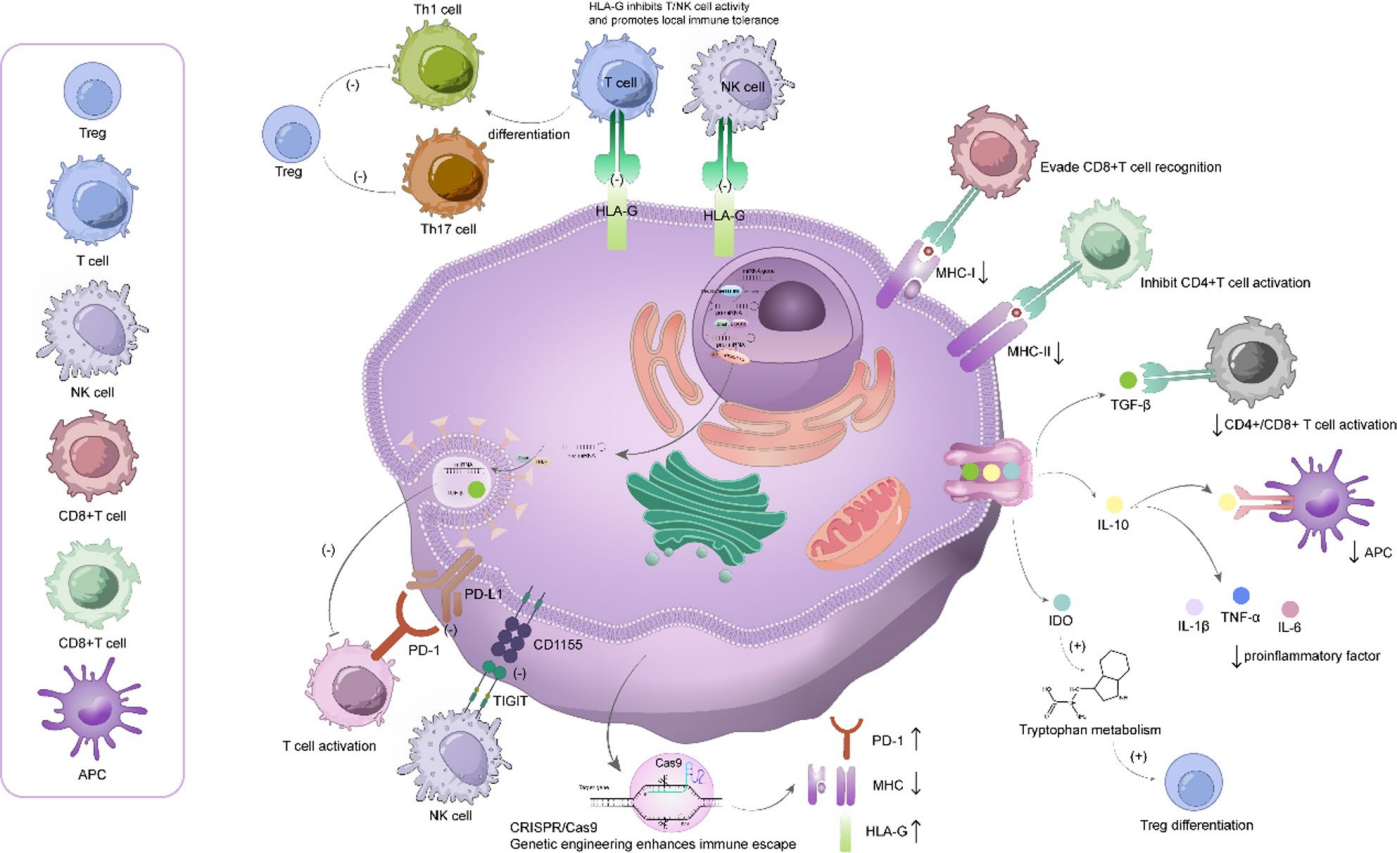
Stem cell immune escape mechanisms

Stem cells possess multiple immune escape and immunoregulatory properties that support graft survival in allogeneic settings [18, 19]. These mechanisms operate at different regulatory levels, including antigen presentation, cytokine signaling, immune checkpoint engagement, and local immune-cell reprogramming. Together, they form the biological basis for subsequent immune engineering and clinical translation strategies (Fig. 2).

Stem cells can escape immune surveillance through multiple strategies. They reduce MHC-I and MHC-II expressions to avoid recognition by CD8⁺ and CD4⁺ T cells. They secrete immunosuppressive cytokines, such as TGF-β and IL-10, which inhibit antigen-presenting cells (APCs) and T cell activation. Indoleamine 2,3-dioxygenase (IDO) promotes tryptophan metabolism, enhancing Treg differentiation and suppressing proinflammatory responses. Stem cells also express immune checkpoint molecules, such as PD-L1 and CD115, which inhibit T and NK cell cytotoxicity through the PD-1 and TIGIT pathways. Expression of HLA-G further suppresses T and NK cell activity and promotes local immune tolerance. CRISPR/Cas9-based gene editing can increase immune evasion by regulating MHC, PD-1, and HLA-G expression. Together, these mechanisms support stem cell survival in immunocompetent environments.

#### Regulation of MHC molecules

Reduction of MHC expression is a key mechanism by which stem cells evade immune recognition. MHC class I and class II molecules present antigens to T cells and initiate adaptive immune responses. Many stem cell types, including mesenchymal stem cells (MSCs) and embryonic stem cells (ESCs), naturally express low levels of MHC molecules. This feature reduces their immunogenicity in allogeneic settings.

Low MHC class I expression limits recognition by CD8⁺ cytotoxic T cells [20–22]. Reduced MHC class II expression further weakens CD4⁺ T-cell activation and downstream immune amplification [23–25]. Together, these properties contribute to the relative immune privilege observed in undifferentiated stem cells.

However, this immune advantage is context-dependent and may not be maintained during differentiation. The immunogenicity of ESCs is closely linked to their developmental state. Undifferentiated ESCs display minimal MHC expression. In contrast, progressive differentiation is accompanied by increased MHC expression. This shift enhances immune recognition and represents a major challenge for long-term graft survival [26–28].

Importantly, these observations highlight a key limitation of MHC-based immune evasion strategies. Complete or sustained loss of MHC class I expression can also trigger natural killer (NK) cell–mediated “missing-self” recognition. Therefore, immune escape through MHC regulation requires a balanced approach rather than simple MHC suppression. This limitation necessitates additional regulatory mechanisms, which are discussed in subsequent sections.

#### Secretion of immunosuppressive cytokines and factors

Stem cells shape immune responses through a coordinated secretome. This secretome includes cytokines, chemokines, growth factors, and metabolic enzymes. These factors suppress immune activation and support immune tolerance in allogeneic environments [29, 30].

At the molecular level, several key mediators directly inhibit immune effector functions. Transforming growth factor-β (TGF-β) suppresses CD4⁺ and CD8⁺ T-cell activation. It also inhibits natural killer (NK) cell cytotoxicity and promotes regulatory T-cell differentiation [31, 32]. Interleukin-10 (IL-10) reduces pro-inflammatory cytokine production and impairs antigen-presenting cell function, thereby limiting T-cell priming [29, 33, 34]. Prostaglandin E₂ (PGE₂) restrains T-cell proliferation and blocks dendritic-cell maturation [35, 36]. Indoleamine 2,3-dioxygenase (IDO) depletes local tryptophan and suppresses activated T cells through metabolic control [37–40]. These mediators are summarized in Table 1.

**Table 1:** Key regulatory pathways promoting immune tolerance in SCT

Mechanism	Key Molecule/Cell	Effect	Outcome	References
TGF-β and IL-10 Pathways	TGF-β, IL-10 (Stem cells)	Stem cells secrete TGF-β and IL-10, promoting Treg generation	Enhances Treg production, leading to immune suppression and tolerance	[41, 42]
IDO Pathway	IDO (Stem cells)	IDO catalyzes tryptophan degradation into kynurenine, suppressing T-cell proliferation	Induces Treg generation and suppresses T-cell proliferation	[43, 44]
PGE2 Pathway	PGE2 (Stem cells)	Stem cells secrete PGE2, inhibiting dendritic cell maturation and T-cell activation	Promotes Treg and M2 macrophage generation, suppressing inflammatory responses	[45, 46]
NO Pathway	NO (Stem cells)	NO secretion by stem cells inhibits T-cell proliferation and dendritic cell maturation	Induces T-cell apoptosis and maintains immune tolerance	[47]

**Fig. 3:**
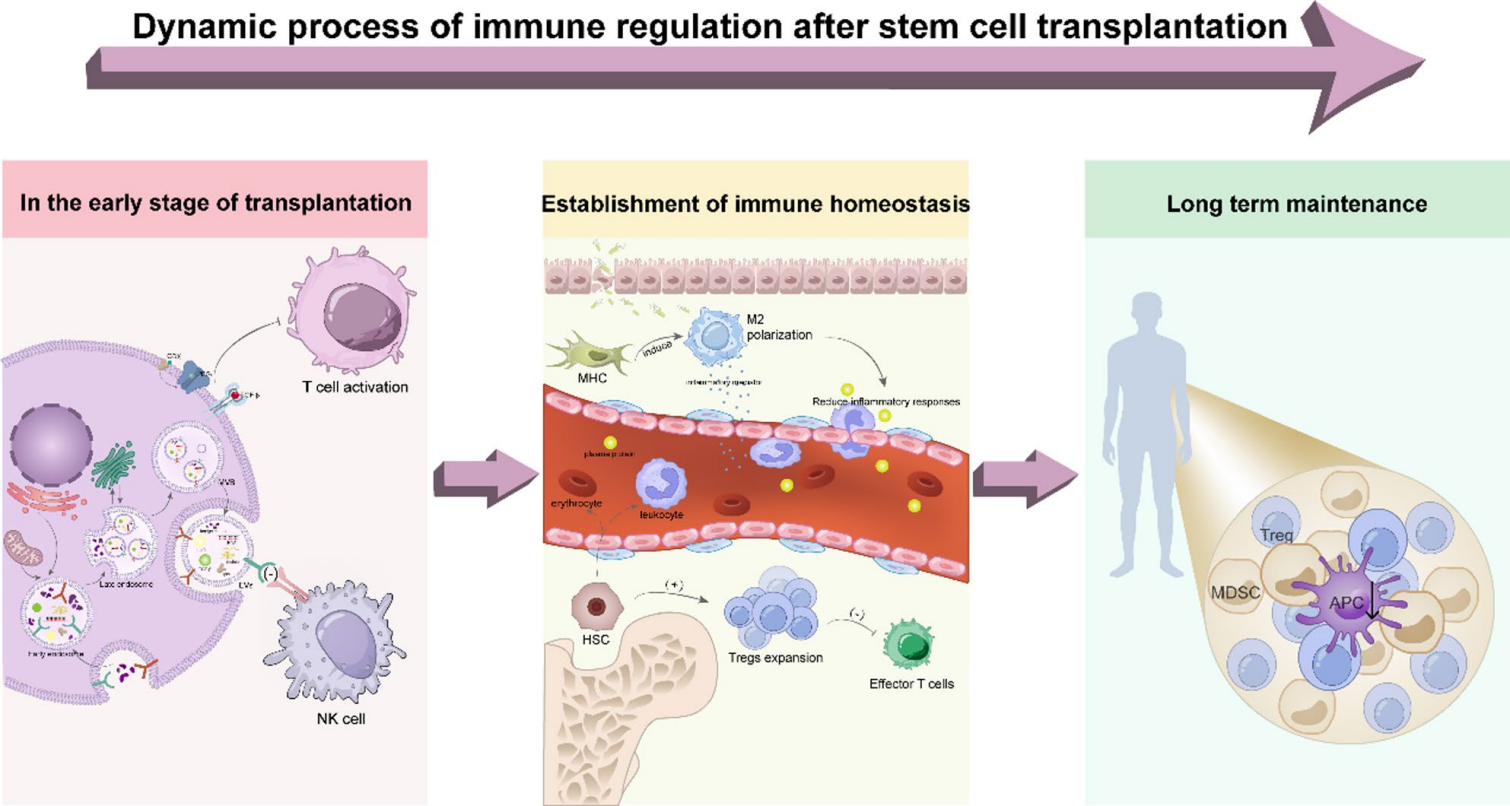
Dynamic process of immune regulation after SCT

Beyond these direct molecular effects, the stem cell secretome induces broad reprogramming of immune cells. Macrophages are polarized toward an anti-inflammatory M2 phenotype. Dendritic-cell maturation is inhibited, which reduces antigen presentation. B-cell activation and antibody production are suppressed. NK-cell proliferation and cytotoxicity are attenuated [23, 25, 48–56]. Through these coordinated actions, stem cells integrate innate and adaptive immune regulation (Fig. 3).

From a translational perspective, this immunomodulatory profile is highly sensitive to environmental cues. In vitro priming with inflammatory cytokines, hypoxia, or three-dimensional culture enhances the secretion of TGF-β, IL-10, PGE₂, and IDO [57–59]. These strategies increase immunosuppressive potency. However, they also introduce variability. Donor heterogeneity and batch-to-batch differences remain major challenges for standardization in clinical applications.

Immune regulation after stem cell transplantation is a dynamic process. In the early stage, stem cells face immune attacks from host T and NK cells. These cells recognize transplanted cells and activate cytotoxic responses. In the middle stage, immune homeostasis is gradually established. Anti-inflammatory factors and immune modulators reduce immune activation. M2 macrophage polarization and Treg expansion help control inflammation. In the long term, a stable immune environment supports the survival and function of transplanted cells.

#### Expression of non-classical HLA molecules (HLA-G and HLA-E) in immune tolerance

Stem cells can evade immune attack by expressing non-classical HLA molecules, mainly HLA-G and HLA-E. These molecules act as natural immune checkpoints and protect grafts from both T-cell– and NK-cell–mediated destruction.

HLA-G binds to inhibitory receptors on T cells, NK cells, and antigen-presenting cells (APCs). This reduces their cytotoxic activity and suppresses antigen presentation. HLA-G also promotes the expansion of regulatory T cells (Tregs) and the differentiation of tolerogenic dendritic cells, which further enhance immune tolerance. High HLA-G expression has been linked with improved outcomes in allogeneic SCT, including lower graft rejection and reduced GVHD [23, 60, 61]. In preclinical and clinical studies, blockade of HLA-G reversed the immunosuppressive effect of MSCs, confirming its role in graft protection [56, 62, 63]. Clinical cohorts also show that soluble HLA-G levels correlate with reduced GVHD and improved survival [64–67].

HLA-E binds the inhibitory receptor NKG2A on NK cells, preventing “missing-self” recognition. Co-expression with PD-L1 in gene-edited stem cells further enhances survival in immunocompetent models by suppressing both NK- and T-cell responses [16, 68, 69].

Together, HLA-G and HLA-E provide a multilayered immune shield that reduces effector cell activation, induces regulatory populations, and improves graft acceptance. These mechanisms lay the foundation for later discussions on microenvironmental reprogramming and exosome-mediated regulation.

### Modulation of the microenvironment

In addition to systemic immune regulation, stem cells actively remodel the local microenvironment at transplantation sites. The graft niche is often the first location where donor cells encounter immune surveillance. Therefore, local immune modulation is critical for preventing early rejection and supporting engraftment [23, 33, 70].

Stem cells promote the accumulation of immunosuppressive immune subsets within the graft niche. Regulatory T cells and myeloid-derived suppressor cells are selectively recruited and expanded. These cells suppress effector T-cell activity and limit NK-cell–mediated cytotoxicity [71–74]. This shift reduces local inflammation and favors tolerance.

Stem cells also regulate antigen-presenting cell function. They inhibit dendritic-cell maturation and macrophage activation. As a result, antigen presentation and T-cell priming are attenuated at the graft site [75, 76]. This local control prevents excessive immune amplification.

Paracrine signaling further stabilizes the tolerogenic microenvironment. Extracellular vesicles serve as key mediators of intercellular communication. Through EV-mediated signaling, stem cells reinforce immune regulation under inflammatory conditions [77–79]. The mechanistic roles of EVs are discussed in detail in Sect. "The Role and Function of Extracellular Vesicles in SCT Immune Regulation".

### The role and function of extracellular vesicles in SCT immune regulation

#### Immunoregulatory roles of extracellular vesicles in SCT

Extracellular vesicles (EVs) are membrane-bound particles released by cells. They carry proteins, lipids, and nucleic acids that reflect the functional state of their parental cells. According to MISEV guidelines, EVs include exosomes, microvesicles, and apoptotic bodies [80]. In SCT, stem cell–derived EVs act as important mediators of immune regulation within the graft microenvironment.

The immunoregulatory effects of EVs are largely determined by their molecular cargo. EVs transport immunosuppressive cytokines, including transforming growth factor-β (TGF-β) and interleukin-10 (IL-10). These cytokines suppress CD4⁺ and CD8⁺ T-cell activation, inhibit natural killer (NK) cell cytotoxicity, and promote the expansion of regulatory T cells (Tregs) [81–84]. Through these actions, EVs contribute to the establishment of immune tolerance at transplantation sites.

In addition to soluble factors, EVs present inhibitory surface molecules that directly modulate immune signaling. Programmed death-ligand 1 (PD-L1) expressed on EV membranes interferes with T-cell receptor signaling and limits effector T-cell activation. This contact-dependent mechanism further strengthens local immune suppression within the graft niche.

EVs also regulate immune responses through non-coding RNAs. Several EV-associated microRNAs act as key regulators of inflammatory signaling. For example, miR-146a and miR-223 inhibit major inflammatory pathways, including NF-κB and STAT signaling. These microRNAs reduce the production of pro-inflammatory cytokines and stabilize immune tolerance in the transplantation microenvironment [85–87].

Through the coordinated delivery of proteins and regulatory RNAs, EVs exert broad immunomodulatory effects on both innate and adaptive immune cells. They suppress T-cell proliferation, induce tolerogenic dendritic cells, inhibit B-cell activation, and promote macrophage polarization toward an anti-inflammatory M2 phenotype [88–93]. These coordinated effects allow EVs to reshape the immune microenvironment without direct cell engraftment, making them an important component of immune regulation in SCT.

#### Biomarker roles of extracellular vesicles in SCT

Beyond their functional roles in immune regulation, EVs also serve as informative biomarkers in SCT. EVs circulate in body fluids and reflect immune status, graft behavior, and ongoing immune interactions. Alterations in EV cargo are associated with immune activation, graft rejection, and graft-versus-host disease (GVHD) [83, 84, 94].

Several studies report correlations between EV-associated microRNAs or immune-related proteins and GVHD severity or treatment response [95, 96]. Compared with soluble biomarkers, EVs provide improved stability and sensitivity, as their cargo is protected by lipid membranes.

However, the clinical application of EV-based biomarkers remains limited. Standardized methods for EV isolation, characterization, and quantification are still lacking. These technical challenges complicate cross-study comparison and delay routine clinical implementation.

#### Mechanistic challenges of EV-mediated immune regulation

Extracellular vesicles avoid risks associated with live-cell transplantation. They do not engraft or proliferate, which reduces concerns related to tumorigenesis and uncontrolled growth [61, 97]. These features make EVs valuable tools for mechanistic studies of immune regulation.

However, EV heterogeneity remains a major limitation. Cargo composition varies with cell source, differentiation state, and culture conditions. Technical variability in isolation and characterization further complicates reproducibility [98–100]. These challenges must be addressed to link mechanistic insights with future translational development [101, 102].

Evidence from experimental models supports the immunomodulatory role of EVs in SCT. In murine graft-versus-host disease (GVHD) models, mesenchymal stem cell (MSC)-derived EVs reduced alloreactive T-cell expansion, increased Treg frequencies, alleviated tissue injury, and significantly improved survival [103–105]. Similar effects were observed in cardiovascular transplantation models, where induced pluripotent stem cell (iPSC)-derived EVs prolonged graft survival by delivering immunosuppressive cytokines and regulatory microRNAs [106].(Table 2).

By transmitting immunoregulatory signals without requiring cellular engraftment, EVs reproduce key immune-modulating functions of stem cells while avoiding risks associated with live-cell transplantation, such as uncontrolled proliferation. These findings establish EVs as functional and biologically active regulators of immune tolerance in SCT.

**Table 2:** Comparative characteristics of EVs derived from different stem cell sources

Source of EVs	Key cargo	Distinctive features	Functional hghlights	Representative references
iPSC-EVs	Pluripotency-associated miRNAs (miR-302/367 cluster), pro-regenerative proteins	Retain features of pluripotency; enriched in molecules regulating self-renewal and immune modulation	Stronger pro-regenerative effects; enhance angiogenesis; potent immunomodulation in transplantation settings	[107] [108]
MSC-EVs	Anti-inflammatory cytokines (TGF-β, IL-10); regulatory miRNAs (miR-146a, miR-21, miR-223)	Rich in immunosuppressive molecules and tissue repair–related factors	Suppress inflammatory responses; inhibit NF-κB signaling; promote macrophage M2 polarization; enhance tissue repair	[100] [109]
ESC-EVs	Developmental regulators, Wnt/β-catenin signaling molecules	Reflect embryonic origin; regulate developmental pathways	Promote tissue regeneration; support cell proliferation and differentiation	[110] [111]

Taken together, stem cell–mediated immune regulation is a multi-layered process that integrates intrinsic immune-evasive properties, active immunosuppressive signaling, microenvironmental modulation, and paracrine communication through extracellular vesicles. These mechanisms do not function in isolation. Instead, their combined and context-dependent actions determine graft survival, immune tolerance, and long-term therapeutic efficacy.

Importantly, while these immune regulatory pathways explain the relative immune privilege of stem cells, they are often insufficient on their own to ensure durable engraftment in clinically complex settings. This limitation has driven the development of genetic engineering, microenvironmental control, and disease-oriented immune optimization strategies. Building upon the mechanistic framework outlined in this chapter, the following sections focus on how these principles are translated into practical applications, clinical interventions, and emerging therapeutic platforms.

## Application of SCT

**Fig. 4:**
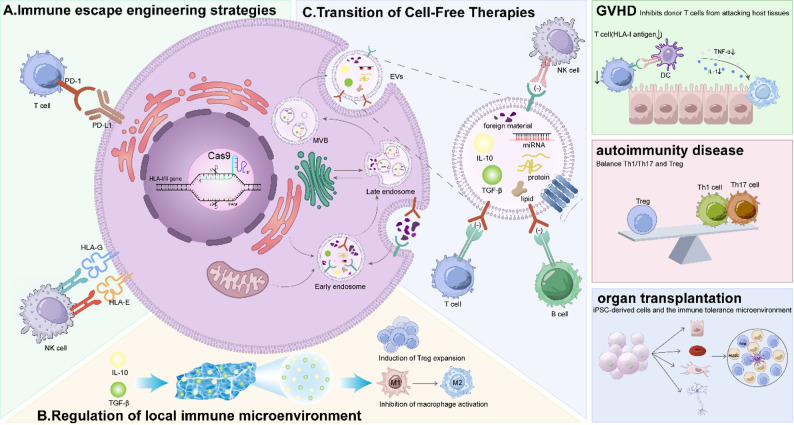
Stem cell therapies use multiple strategies to control immune responses. (**A**) Gene editing technologies, such as CRISPR/Cas9, help stem cells evade immune detection by modifying HLA genes and increasing checkpoint expression (e.g., PD-L1, HLA-G). (**B**) Stem cells regulate the local immune microenvironment by releasing IL-10 and TGF-β, which induce Treg expansion and suppress macrophage activation. (**C**) Cell-free therapies, including EVs, carry immunomodulatory molecules like miRNAs, proteins, and lipids. These molecules reduce immune activation and promote tolerance

Building upon the immune regulatory mechanisms outlined in Chap. 1, this section focuses on how these principles are translated into practical stem cell transplantation (SCT) strategies across different disease contexts (Fig. 4). Rather than describing immune modulation as an abstract biological phenomenon, Chap. 2 emphasizes its application-oriented implementation through engineered cell products, clinically validated immunomodulatory approaches, and disease-specific therapeutic designs.

In graft-versus-host disease (GVHD), stem cell–based immune modulation primarily aims to attenuate early donor T cell activation while preserving overall immune competence. In autoimmune diseases, therapeutic strategies focus on restoring immune homeostasis by rebalancing pro-inflammatory and regulatory immune cell subsets, including Th1, Th17, and regulatory T cells. In solid organ transplantation and regenerative medicine, immune-modulated iPSC-derived products are increasingly combined with local immune conditioning to support long-term graft acceptance and tissue repair.

Collectively, these applications illustrate how immune escape and immune regulation strategies evolve from mechanistic concepts into clinically actionable interventions. This translational progression forms the foundation for enabling technologies and future innovations discussed in subsequent chapters.

### Translational application of immune escape strategies

#### Gene-edited stem cell products for disease-oriented applications

Immune rejection remains a major barrier to the clinical translation of stem cell transplantation. To address this challenge, genetic engineering is increasingly applied to generate immune-evasive stem cell products designed for specific disease indications, rather than as generalized immune-modulatory tools.

In translational studies, induced pluripotent stem cells (iPSCs) are the most frequently engineered cell type due to their scalability and differentiation potential. Deletion of classical HLA class I and class II molecules using CRISPR/Cas9 or TALEN platforms reduces alloreactive T-cell recognition in allogeneic settings [112]. To prevent natural killer cell–mediated clearance, non-classical HLA molecules such as HLA-G or HLA-E are selectively retained or reintroduced. These strategies have been successfully applied to iPSC-derived therapeutic cell products, resulting in prolonged graft persistence in immunocompetent animal models [113, 114].

Beyond HLA modulation, immune checkpoint engineering further supports translational application. Overexpression of programmed death-ligand 1 (PD-L1) suppresses T-cell activation, while CD47 expression limits macrophage-mediated phagocytosis. These modifications are often combined to generate hypoimmunogenic or so-called universal donor stem cell products. In vivo studies demonstrate reduced immune clearance and sustained graft survival without continuous systemic immunosuppression (Table 3) [115].

**Table 3:** Key immune evasion mechanisms in SCT

Mechanism	Key Molecule/Cell	Effect	Outcome	References
PD-1/PD-L1 Pathway	PD-L1 (Stem cells), PD-1 (T cells)	Stem cells express PD-L1, binding to PD-1 on T cells, inhibiting T-cell activation and proliferation	Suppresses T-cell mediated immune responses, inducing T-cell dysfunction or apoptosis	[16]
CTLA-4/CD80/CD86 Pathway	CTLA-4 (Stem cells/Tregs), CD80/CD86 (APCs)	CTLA-4 on stem cells or Tregs binds CD80/CD86 on APCs, inhibiting T-cell co-stimulation	Blocks full activation of T cells, inhibiting immune response	[116]
Tim-3/Galectin-9 Pathway	Tim-3 (Stem cells), Galectin-9 (T cells)	Tim-3 on stem cells binds Galectin-9 on T cells, inhibiting Th1 and CTL activity	Induces T-cell apoptosis, inhibits Th1 and CTL mediated immune response	[117]
LAG-3/MHC-II Pathway	LAG-3 (Stem cells), MHC-II (APCs)	LAG-3 on stem cells binds MHC-II on APCs, inhibiting T-cell activation	Inhibits T-cell proliferation and function, promotes immune tolerance	[118]
VISTA Pathway	VISTA (Stem cells)	Stem cells express VISTA, suppressing T-cell activation and proliferation	Maintains immune tolerance and inhibits T-cell mediated responses	[119]

Importantly, these immune-evasive strategies are applied in a disease-specific manner. Gene-edited iPSC-derived pancreatic β cells restore insulin production and resist immune-mediated destruction in type 1 diabetes models [120–122]. In cardiovascular disease, universal donor iPSC-derived cardiomyocytes show improved engraftment and functional integration after myocardial injury, with reduced reliance on immunosuppressive drugs [123, 124]. In autoimmune disease models, including multiple sclerosis and rheumatoid arthritis, engineered stem cells exert therapeutic effects primarily through immune modulation rather than long-term tissue replacement [125–127].

Collectively, these studies demonstrate that genetic engineering enables immune escape strategies to be translated into application-oriented stem cell products. The focus has shifted from mechanistic immune evasion to disease-adapted design with defined in vivo outcomes.

#### Precision editing platforms supporting clinical translation

CRISPR/Cas9 is currently the most widely used genome-editing platform for engineering immune-evasive stem cells because of its high efficiency and flexible design. In regenerative medicine research, this system is mainly used to modify immune-related loci and introduce regulatory elements that enable allogeneic cell transplantation.

However, conventional CRISPR/Cas9 editing introduces double-strand DNA breaks. These breaks may cause off-target mutations, chromosomal rearrangements, and genomic instability. Such alterations can increase long-term safety concerns, including the risk of malignant transformation after transplantation [128].

To address these limitations, new precision editing platforms have been developed to reduce genome damage during editing. Adenine base editors, such as adenine base editor 8e (ABE8e), allow single-nucleotide conversion without generating double-strand breaks. This mechanism significantly reduces chromosomal rearrangements and improves genomic stability compared with conventional CRISPR editing [129].

Prime editing further expands this strategy. It enables targeted insertions, deletions, and base substitutions without donor DNA templates and without introducing double-strand breaks. By minimizing DNA damage during editing, prime editing provides a safer strategy for modifying immune-related genes in stem cells.

These precision editing platforms are therefore increasingly viewed as important tools for reducing tumorigenic risks associated with genome engineering. By limiting DNA break–induced genomic instability, they help improve the long-term safety of engineered stem cells intended for clinical transplantation.

In parallel, chemical reprogramming has emerged as a virus-free strategy for generating immune-compatible stem cells. Chemically induced pluripotent stem cells (CiPSCs) are generated using defined small molecules that regulate key signaling pathways. This approach avoids viral integration and reduces insertional mutagenesis, which may also contribute to improved genomic safety during stem cell production [130].

Overall, advances in precision genome editing and chemical reprogramming are shifting immune engineering toward safer and more controllable platforms. Maintaining genomic stability while achieving effective immune evasion remains a key requirement for the clinical translation of engineered stem cells [131–133].

#### Engineering the local immune microenvironment

In addition to intrinsic genetic modification, external engineering strategies are increasingly used to modulate the immune microenvironment at the transplantation site. Unlike the cell-intrinsic immune mechanisms discussed in Chap. 1, these approaches aim to achieve spatially restricted immune regulation through localized delivery systems.

Local administration of immunosuppressive cytokines, such as transforming growth factor-β and interleukin-10, suppresses effector T-cell and macrophage activation while promoting regulatory T-cell differentiation [132, 134–136]. When delivered directly to the graft site, these factors enhance immune tolerance without inducing systemic immunosuppression.

Recent advances in biomaterials enable controlled and sustained cytokine release. Cytokine-loaded scaffolds and hydrogel platforms can be co-implanted with stem cells to establish a transient immunoregulatory niche. In preclinical transplantation models, these systems improve graft persistence and reduce immune-mediated complications, including graft-versus-host disease [137, 138].

Microenvironment engineering functions as a complementary strategy rather than a replacement for genetic immune evasion. By confining immune modulation to the graft site, these approaches preserve systemic immune competence while enhancing local graft protection. This localized control improves translational feasibility and clinical safety.

### Advancing immune regulation in clinical settings

#### Established clinical applications of stem cell–mediated immunomodulation

While genetic and microenvironmental engineering strategies provide foundational immune control, stem cell–based immunomodulation has already entered clinical practice in several disease settings. In these contexts, stem cells are primarily used as active therapeutic agents to attenuate excessive immune responses and restore immune balance, rather than as passive grafts.

The most established clinical application of stem cell–mediated immune regulation is the treatment of steroid-refractory graft-versus-host disease (GVHD). In this setting, intravenously infused mesenchymal stem cells (MSCs) reduce systemic inflammation, suppress alloreactive T-cell expansion, and limit immune-mediated tissue injury. Based on consistent clinical benefit, the allogeneic MSC product remestemcel-L (RYONCIL) has been approved in the United States for pediatric patients with steroid-refractory acute GVHD [139].

Beyond GVHD, stem cell therapies have been evaluated in autoimmune and inflammatory diseases characterized by immune dysregulation. Clinical studies in Crohn’s disease, systemic lupus erythematosus, and rheumatoid arthritis report reduced inflammatory activity and partial restoration of immune homeostasis following MSC administration [140–142]. These outcomes highlight the translational value of stem cell–based immune modulation, particularly in patients who respond poorly to conventional immunosuppressive therapies.

Importantly, in these clinical contexts, stem cells function as transient immune regulators rather than long-term engrafting cells. This distinction supports their safety profile and facilitates regulatory acceptance.

#### Checkpoint-enhanced stem cells in clinical contexts

Recent translational strategies aim to enhance immune tolerance by incorporating immune checkpoint modulation into stem cell therapies. Rather than re-defining checkpoint biology, these approaches focus on improving graft persistence and therapeutic durability in vivo.

Overexpression of immune inhibitory molecules such as programmed death-ligand 1 (PD-L1) has been shown to reinforce local immune suppression and reduce alloreactive T-cell activation in transplantation-relevant settings [140, 142, 143]. In preclinical and early translational studies, PD-L1–enhanced MSCs prolonged immune tolerance without inducing systemic immunosuppression or overt immune escape.

Checkpoint pathways related to cytotoxic T-lymphocyte–associated protein 4 (CTLA-4) have also been explored. CTLA-4–based strategies attenuate T-cell co-stimulation and reduce immune-mediated rejection in transplantation models [18, 144, 145]. In translational settings, these approaches are positioned as adjunctive tools to improve efficacy and safety, rather than as replacements for established immunotherapies.

Together, checkpoint-enhanced stem cell products represent a pragmatic extension of immune regulation strategies into clinically relevant frameworks.

#### Integration of stem cell therapy with advanced immunotherapies

Stem cell–based immune modulation is increasingly explored in combination with advanced immunotherapies to manage treatment-limiting immune toxicity. Chimeric antigen receptor T (CAR-T) cell therapy has achieved substantial clinical success in hematologic malignancies but is frequently complicated by cytokine release syndrome and immune-mediated tissue injury [140, 142, 143].

In this context, MSCs have been investigated as supportive cellular therapies to stabilize the immune environment during CAR-T treatment. Preclinical studies and early clinical observations suggest that MSC co-administration reduces excessive inflammation, mitigates immune-related adverse events, and supports treatment tolerability [18, 144, 145].

Importantly, this combinatorial strategy does not compromise the antitumor efficacy of CAR-T cells. Instead, it highlights the flexibility of stem cell–based immune regulation as an enabling platform to improve the safety profile of emerging immunotherapies [142, 145].

To summarize representative immune-modulatory strategies currently in clinical or advanced preclinical development, Table 4 provides an overview of therapeutic approaches, disease indications, development stages, and key outcomes.

**Table 4:** Clinical evidence summary of immune evasion and modulation strategies

Mechanism	Clinical Phase	Key Findings	References
PD-1/PD-L1 Axis	Phase I Trial	PD-L1-overexpressing MSCs reduced Grade III-IV GVHD incidence by 60% with no tumorigenesis reported	[146]
IDO/Tryptophan Metabolism	Phase II Trial	IDO+ MSCs reduced T-cell activation by 50% in steroid-refractory Crohn’s disease patients	[139, 147]
HLA-G/HLA-E Retention	Preclinical Study	CRISPR-edited iPSCs achieved 80% survival in immunocompetent mice without NK cell activation	[16]
MSC-Derived Exosomes	Phase II Trial	ExoFlo™ (MSC exosomes) reduced GVHD severity by 40% within 28 days in steroid-refractory patients	[148]
Dual Checkpoint (PD-L1 + TIGIT)	Preclinical Study	Engineered MSCs reduced acute rejection rates by 75% in murine cardiac transplant models	[138]

### Disease-specific applications of stem cell therapies

Stem cell–based therapies have been increasingly explored for immune-related diseases characterized by chronic inflammation and dysregulated immune activation. Rather than acting as universal immune suppressors, stem cells are adapted to disease-specific contexts where immune imbalance directly drives tissue injury or graft failure. This section summarizes representative disease-oriented applications, with emphasis on clinical relevance and therapeutic outcomes.

#### Prevention of graft-versus-host disease

Graft-versus-host disease (GVHD) remains one of the most severe and life-threatening complications of allogeneic stem cell transplantation. It is driven by donor-derived immune cells that recognize host tissues as foreign, leading to systemic inflammation and multi-organ injury. Despite improvements in pharmacologic immunosuppression, GVHD is still associated with substantial morbidity, high infection risk, and treatment-related toxicity.

In this disease context, stem cell–based strategies for GVHD prevention are designed to attenuate early alloimmune activation while preserving overall immune competence, rather than inducing sustained global immunosuppression. Induced pluripotent stem cell (iPSC)–derived immunomodulatory products provide a scalable and controllable platform to meet these requirements. Their therapeutic goal is to transiently reshape the inflammatory milieu during the initiation phase of GVHD, which is a critical determinant of disease severity and progression.

Preclinical GVHD models demonstrate that iPSC-derived immunomodulatory cell products suppress pathogenic donor T-cell expansion, reduce systemic inflammatory cytokine release, and promote regulatory immune populations during early disease development [144, 149]. Importantly, these effects are achieved without prolonged systemic immune suppression, addressing a major limitation of current GVHD prophylactic regimens.

Safety control is particularly critical in GVHD prevention, as excessive or persistent immune suppression increases susceptibility to opportunistic infections and disease relapse. To mitigate these risks, disease-adapted regulatory circuits have been incorporated into iPSC-based platforms. Inducible expression systems allow immunomodulatory activity to be restricted to inflammatory conditions, thereby limiting off-target immune suppression. In parallel, suicide gene strategies, such as herpes simplex virus thymidine kinase (HSV-TK) modules, enable selective elimination of transplanted cells in the event of adverse immune effects or uncontrolled persistence [150–152].

Advances in scalable differentiation protocols and bioreactor-based manufacturing further support the translational feasibility of iPSC-derived immunomodulatory cells for GVHD prevention [153–156]. Collectively, these disease-tailored strategies illustrate how stem cell–based immune regulation can be specifically adapted to the unique clinical and safety constraints of GVHD, balancing therapeutic efficacy with rigorous control.

#### Stem cell therapies in autoimmune diseases

Autoimmune diseases are characterized by persistent immune activation against self-antigens, resulting in chronic inflammation and progressive tissue damage. Representative conditions include systemic lupus erythematosus, rheumatoid arthritis, and multiple sclerosis [157, 158]. Conventional therapies rely on long-term immunosuppression and are often limited by incomplete disease control and cumulative toxicity.

Stem cell–based therapies offer an alternative approach by restoring immune balance rather than broadly suppressing immune function. Clinical and translational studies report reduced disease activity and improved immune regulation following stem cell administration in several autoimmune conditions [25, 159–161]. These benefits are associated with sustained attenuation of inflammatory responses and enhancement of regulatory immune pathways.

Importantly, several studies suggest that stem cell therapy may reduce dependence on prolonged pharmacologic immunosuppression, thereby improving long-term safety and quality of life [161]. These findings support the role of stem cells as immune-modulatory interventions in selected autoimmune diseases.

#### Stem cells in solid organ transplantation

Immune-mediated rejection remains a central limitation in solid organ transplantation, necessitating lifelong immunosuppressive therapy. While effective in preventing acute rejection, chronic immunosuppression increases the risk of infection, malignancy, and metabolic complications.

Stem cells have been investigated as adjunctive therapies to promote immune tolerance and reduce immunosuppressive burden in organ transplantation. Preclinical and early clinical studies indicate that stem cell co-administration attenuates alloimmune activation and supports immune homeostasis at the graft interface [162, 163]. In kidney, liver, and heart transplantation models, these effects translate into prolonged graft survival and reduced reliance on conventional immunosuppressive drugs [71].

Although large-scale clinical validation is still required, these findings support stem cells as complementary tools to refine immune management in solid organ transplantation.

#### Immune modulation in regenerative medicine

Beyond immune-mediated diseases, stem cells play a critical role in regenerative medicine by integrating immune regulation with tissue repair. This dual function is particularly relevant in conditions where inflammation and tissue injury coexist, such as heart failure, diabetes, and spinal cord injury.

In regenerative settings, stem cells attenuate excessive inflammatory responses while supporting angiogenesis, limiting fibrosis, and promoting structural repair [157, 160]. This coordinated regulation creates a permissive microenvironment for tissue regeneration and functional recovery.

The ability of stem cells to couple immune modulation with regenerative processes distinguishes them from purely cell-replacement or pharmacologic approaches and underlies their broad applicability across complex disease settings.

Despite encouraging clinical progress across diverse disease settings, current applications of immune-modulated stem cell transplantation remain constrained by limitations in precision, scalability, and long-term control. Most existing strategies rely on predefined immune-regulatory designs that may not fully account for inter-patient variability, dynamic immune responses, or complex graft–host interactions in vivo. In addition, the increasing complexity of engineered stem cell products poses challenges for reproducibility, manufacturing standardization, and safety monitoring.

These limitations underscore the need for enabling technologies that can enhance controllability, predictability, and clinical robustness without expanding nonspecific immune suppression. Advances in gene editing, tissue engineering, scalable manufacturing platforms, and artificial intelligence offer new opportunities to refine immune-modulated SCT at both the product and system levels. The integration of these technologies, which is discussed in the following chapter, represents a critical step toward more precise, efficient, and clinically sustainable stem cell–based therapies.

## The future prospects of SCT

While immune-modulated stem cell transplantation has demonstrated therapeutic potential across multiple disease settings, its broader clinical translation remains limited by challenges in precision, reproducibility, scalability, and long-term control. As highlighted in the preceding chapter, current applications often rely on predefined immune-regulatory strategies that may not fully capture patient-specific immune dynamics or complex graft–host interactions. Moreover, increasing product complexity places additional demands on manufacturing consistency and safety assurance.

Against this background, the future development of SCT is increasingly driven by enabling technologies rather than the discovery of new immune mechanisms. Advances in gene editing, tissue engineering, scalable manufacturing systems, and artificial intelligence provide tools to improve controllability, standardization, and predictive capacity throughout the iPSC workflow. This chapter focuses on how these technologies support next-generation SCT by addressing translational bottlenecks and enabling more precise, efficient, and clinically sustainable immune-modulated stem cell therapies.

### Enabling technologies for next-generation SCT

Rather than introducing new immune mechanisms, the future of stem cell transplantation is driven by the integration of enabling technologies that collectively improve precision, reproducibility, and clinical scalability. Gene editing, tissue engineering, advanced manufacturing, and artificial intelligence have each matured independently. Their combined and coordinated application now defines the next phase of iPSC-based SCT development, addressing persistent translational barriers in a system-level manner [164].

#### Gene editing as a tool for standardization and control

In next-generation SCT, gene editing functions as a foundational standardization layer rather than a standalone immune-modulatory innovation. Precisely defined genetic architectures established by CRISPR-based systems reduce inter-batch variability and enable the generation of uniform iPSC-derived products suitable for downstream integration with tissue engineering and manufacturing pipelines [165–169].

Importantly, predictable genetic configurations also facilitate regulatory assessment. When combined with controlled differentiation and structured graft design, gene editing supports more consistent post-transplant behavior, reducing uncertainty related to immune responses and long-term safety.

#### Tissue engineering and structured graft design

Tissue engineering technologies provide spatial and architectural control that complements genetic standardization. Three-dimensional bioprinting and scaffold-based systems enable reproducible graft structures that integrate engineered cells into defined microenvironments [170, 171].

Within an integrated SCT framework, structured graphs serve not only as therapeutic constructions but also as translational platforms. They allow immune–tissue interactions to be evaluated under controlled spatial conditions, improving the alignment between preclinical testing and in vivo performance.

#### Scalable manufacturing and quality control platforms

The clinical implementation of integrated SCT strategies requires manufacturing platforms capable of maintaining consistency across genetically defined and structurally engineered products. Advances in bioreactors, automated processing, and quality control systems support large-scale iPSC expansion and differentiation under standardized conditions [172, 173].

By reducing process-related variability, these platforms enhance the reliability of immune outcomes after transplantation. Manufacturing integration is therefore essential for translating multi-component SCT strategies into clinically reproducible and regulatory-compliant therapies.

### AI-driven precision and personalization in SCT

Artificial intelligence functions as an analytical and predictive framework in stem cell transplantation (SCT). AI does not introduce new biological mechanisms. Instead, it improves efficiency, reproducibility, and decision-making across the iPSC workflow. AI is now used to guide data interpretation, reduce manual bias, and support clinical decisions in regenerative medicine and SCT research [174, 175].

#### AI-assisted iPSC reprogramming and lineage conversion

iPSC reprogramming involves complex transcriptional changes and low efficiency. Machine learning models analyze large gene expressions and imaging datasets to identify optimal factor combinations and culture conditions. These models reduce trial and error and increase reprogramming success rates. AI can also monitor colony morphology and cell quality automatically, which improves consistency and reduces subjective bias in large-scale iPSC production [176, 177].

AI also supports direct lineage conversion. Deep learning models integrate multi-omics and phenotypic data to predict effective conversion strategies for specific cell types. For example, predictive models have been used to improve differentiation of iPSCs into cardiomyocytes or neurons by identifying key signals that drive lineage outcomes [178]. Non-invasive AI tools are also emerging for automated maturity assessment of iPSC-derived cells before transplantation, improving readiness for functional and clinical testing [179–181].

#### AI in gene editing design and safety prediction

AI-based algorithms are now widely used in gene editing design. These models predict editing efficiency and off-target risk for CRISPR and newer editing platforms. AI tools guide selection of guided RNAs and identify genomic contexts that may lead to unintended effects. Such tools improve precision and safety of immune-engineering strategies, which is particularly critical for complex modifications in SCT products [182, 183].

Furthermore, AI helps integrate large datasets from whole-genome sequencing and chromatin maps to refine editing strategies and reduce genomic instability. This supports safer translation of gene-edited cells from bench to clinic by minimizing off-target risks and improving control over engineered immune-regulatory circuits.

#### AI-based drug screening and outcome prediction

AI-driven platforms accelerate drug screening for stem cell applications. These systems analyze large chemical libraries and phenotypic readouts to identify compounds that regulate stem cell survival, differentiation, or immunomodulatory capacity. Deep learning-based image analysis can detect subtle drug effects on iPSC-derived cells, including cardiomyocytes, and classify toxic versus non-toxic responses with high accuracy [184, 185].

AI also enables predictive modeling of therapeutic outcomes. By integrating patient molecular profiles, cell phenotype data, and clinical history, models estimate engraftment potential, differentiation efficiency, and treatment response before transplantation. Predictive modeling helps stratify patients likely to benefit from specific therapy designs, thus advancing personalized SCT strategies [186, 187].

AI may also support optimization of manufacturing and quality control. Algorithms monitor process-critical variables in real time and detect anomalies in cell culture, which helps ensure consistent product quality and safety for clinical use [188–190].

#### AI in immune monitoring and safety management

Beyond cell production and editing, AI supports immune monitoring. Machine learning models interpret high-dimensional data such as flow cytometry, single-cell sequencing, and spatial transcriptomics to identify early signs of rejection or tolerance patterns after SCT. These applications allow clinicians to tailor immunomodulation schedules and reduce adverse outcomes [191].

### Emerging directions and remaining challenges in immune-modulated SCT

**Fig. 5:**
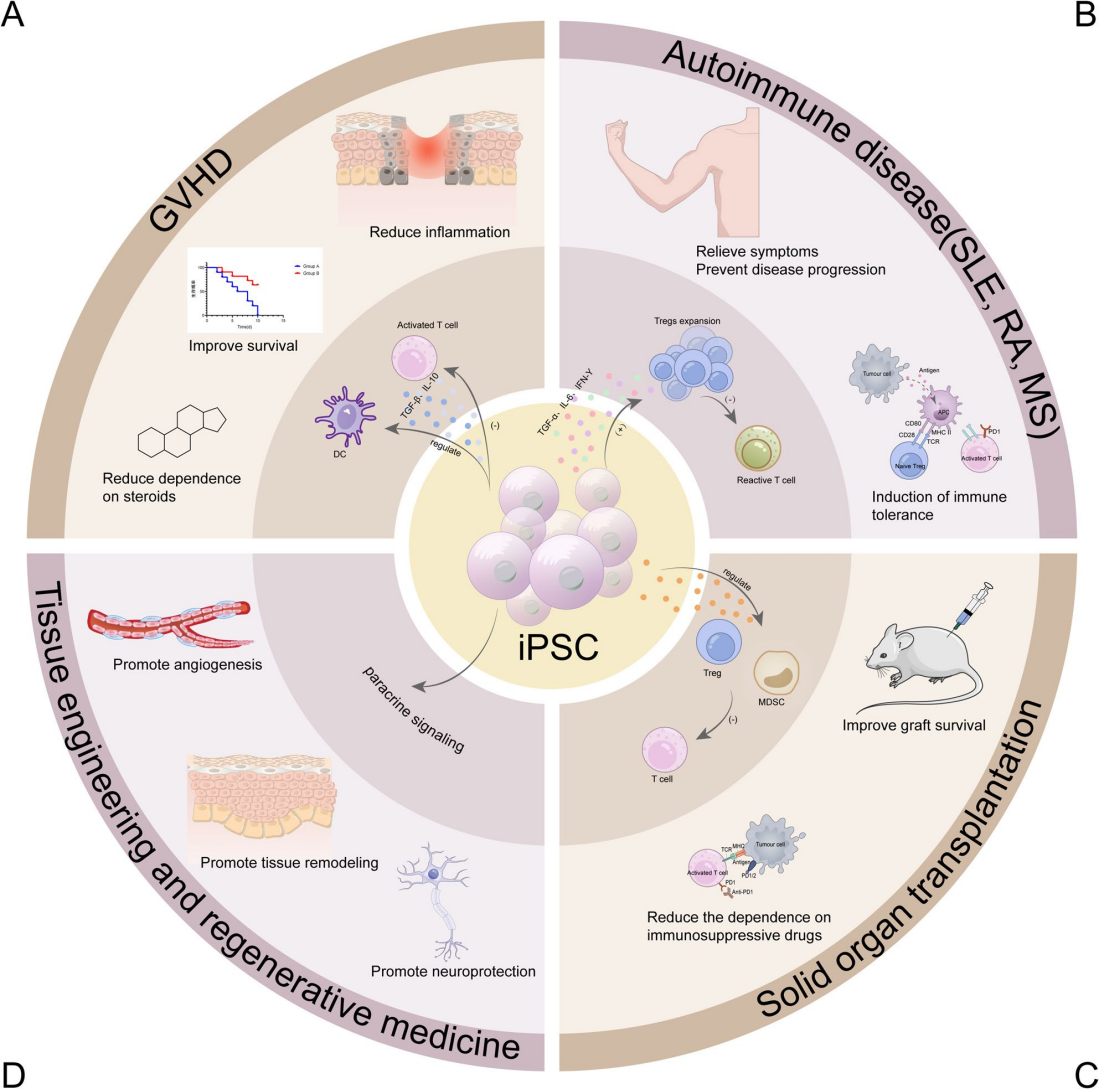
Future directions in immune escape and immune regulation in SCT

Despite progress in immune modulation, major challenges remain. These include long-term safety, controllability, and clinical scalability. Future SCT strategies must focus on precision rather than expanded immune suppression [8, 192] (Fig. 5).

(A) Graft-versus-host disease (GVHD). iPSCs secrete anti-inflammatory cytokines that reduce tissue inflammation in target organs. Treated animals show prolonged survival and a decreased need for systemic steroids.
(B) Autoimmune diseases (SLE, RA, MS). iPSC-derived factors promote expansion of regulatory T cells (Tregs). Expanded Tregs suppress autoreactive T cells and induce long-term immune tolerance. This effect alleviates clinical symptoms and slows disease progression.
(C) Solid organ transplantation. iPSCs increase populations of Tregs and MDSCs. These cells inhibit effector T cell activity and improve graft acceptance in preclinical models. As a result, recipients require lower doses of immunosuppressive drugs.
(D) Tissue engineering and regenerative medicine. Paracrine signals from iPSCs stimulate angiogenesis and enhance tissue remodeling at injury sites. They also support neuroprotection and neural repair in injured tissues.

Central insect. Human iPSCs release a spectrum of growth factors and cytokines. These factors act locally by paracrine signaling to modulate immune responses and promote tissue regeneration.

#### Context-dependent immune modulation and safety control

Continuous expression of immune modulators can improve graft survival but may impair stem cell function and host immunity. Long-term immune checkpoint activation is associated with metabolic stress and increased infection risk [193].

To address this issue, inducible and context-dependent expression systems are being developed. Drug-responsive promoters and inflammation-sensitive genetic switches restrict immune modulation to critical periods, such as early engraftment [194, 195].

Prolonged immune suppression also increases the risk of viral reactivation, including EBV and CMV [196–198]. Future SCT designs must therefore integrate immune modulation with built-in safety controls and post-transplant monitoring.

#### Cell-free and scalable therapeutic alternatives

Cell-free therapies represent an emerging alternative to live cell transplantation. iPSC-derived exosomes provide immunomodulatory effects without risks related to uncontrolled proliferation or long-term engraftment [199–201].

Exosome-based therapies offer advantages in scalability and storage. iPSCs provide a renewable and standardized source of extracellular vesicles, facilitating large-scale production and regulatory approval [202, 203].

However, key challenges remain. These include standardization of isolation methods, control of cargo composition, and long-term efficacy assessment [250].

#### Precision immune monitoring and translational optimization

Future SCT relies on high-resolution immune monitoring technologies. Single-cell RNA sequencing, T cell receptor sequencing, and spatial transcriptomics enable detailed analysis of immune states at graft sites.

These tools support early detection of rejection signals and tolerance-associated profiles. They also allow comparison of different SCT strategies at single-cell resolution [198].

Integration of precision monitoring into clinical workflows will be essential. Data-driven optimization improves safety, supports personalized immune modulation, and accelerates clinical translation.

## The challenges

Despite substantial advances in immune regulation, enabling technologies, and translational optimization, stem cell transplantation (SCT) continues to face several unresolved challenges that limit its widespread and sustainable clinical adoption. As highlighted in the preceding chapters, increasingly sophisticated immune-evasion strategies, gene-editing platforms, and AI-assisted approaches have improved graft persistence and therapeutic precision. However, these innovations also introduce new layers of complexity related to safety, manufacturing robustness, immune balance, and regulatory oversight.

This chapter focuses on the key challenges that must be addressed to ensure the long-term effectiveness and clinical reliability of immune-modulated SCT. Rather than reiterating specific immune mechanisms or technological advances discussed earlier, the following sections critically examine safety concerns, scalability and standardization barriers, the delicate balance between immune suppression and host protection, limitations in personalized implementation, and ethical and regulatory constraints. Together, these challenges define the boundaries within which next-generation SCT strategies must be optimized to achieve durable, safe, and globally applicable therapeutic outcomes.

### Safety and long-term effectiveness

Enhancing immune evasion through genetic modification or advanced engineering strategies raises important safety concerns. Uncontrolled proliferation and malignant transformation remain potential risks, especially for long-lived or permanently engrafted cells [204, 205]. Rigorous preclinical testing and long-term clinical monitoring are therefore essential to detect rare but severe adverse events [206, 207].

In addition to tumorigenic risk, immune evasion strategies may affect stem cell function and differentiation capacity. Reduced expression of MHC molecules or sustained immune checkpoint signaling can alter cell–environment interactions that are important for normal maturation and tissue integration [193, 208, 209]. These functional changes may compromise long-term therapeutic efficacy, even when short-term graft survival is improved.

The durability of immune protection also remains uncertain. Long-term graft acceptance must be achieved without disrupting systemic immune homeostasis or increasing susceptibility to delayed complications. Extended follow-up studies are required to evaluate graft function, immune balance, and late-onset adverse effects [71, 210].

### Manufacturing and scalability

Large-scale production of stem cell–based therapies presents major technical and regulatory challenges [211]. Genetic and epigenetic stability must be maintained during prolonged culture and expansion. Manufacturing processes must also comply with stringent and often region-specific regulatory standards.

Standardization is particularly difficult for complex products, including gene-edited cells and exosome-based therapies. Variability in production protocols can lead to inconsistent quality and efficacy [211, 212]. These limitations reduce reproducibility and increase manufacturing costs, which remain major barriers to widespread clinical implementation.

### Balancing immune suppression and host protection

Successful transplantation requires effective suppression of alloimmune responses while preserving host immune defense. Excessive immune suppression increases the risk of opportunistic infections and malignancies. In contrast, insufficient suppression results in graft rejection and inflammatory injury [213, 214].

Immune evasion strategies, such as reduced antigen presentation or immune checkpoint modulation, highlight this trade-off. While these approaches improve graft tolerance, they may impair immune surveillance and increase vulnerability to viral reactivation or tumor development [140, 215, 216]. This risk is particularly relevant in patients with pre-existing immune dysfunction or prolonged immunosuppressive exposure.

Achieving an appropriate balance remains challenging in heterogeneous patient populations. Immune status can change over time due to infection, inflammation, or disease progression. These dynamics complicate dose selection and treatment duration, underscoring the need for adaptable and closely monitored immunomodulatory strategies [217, 218].

### Personalization and precision medicine

Personalized stem cell therapies aim to align immune modulation strategies with individual patient immune profiles. Advances in immune profiling, including single-cell sequencing and high-throughput assays, improve understanding of patient-specific immune responses. These tools support rational selection of cell sources and immunomodulatory approaches [219].

However, clinical implementation remains limited. High costs, complex logistics, and restricted access to advanced diagnostic platforms hinder routine application. In addition, predictive computational models require further validation before they can reliably guide clinical decision-making in diverse patient populations.

### Ethical and regulatory challenges

The clinical translation of stem cell therapies raises significant ethical and regulatory challenges. Genome editing improves immune compatibility but introduces concerns related to unintended genetic alterations and unknown long-term consequences. Regulatory agencies therefore require comprehensive evaluation of genomic stability and off-target effects.

Access to advanced therapies remains uneven across regions. High production costs and specialized infrastructure limit availability in low-resource settings. Regulatory differences between countries further complicate global clinical adoption.

Recent regulatory guidance reflects increasing caution. Stringent genomic analyses are required for gene-edited products. In Europe, ethical considerations surrounding universal donor iPSCs currently favor autologous or alternative cell sources under existing regulatory frameworks [220].

## Conclusion

Immune escape and regulation are crucial for successful stem cell therapies. They minimize rejection and manage inflammation, particularly in allogeneic transplantation. Current advances include CRISPR gene editing, exosome therapy, and immune checkpoint modulation. These methods enhance graft survival and support long-term immune tolerance. Emerging strategies apply to GVHD, autoimmune disorders, and organ failure. CRISPR-edited iPSCs offer potential for universal donor cells. Exosome therapies have reduced GVHD incidence in clinical trials. AI supports immune response prediction to optimize treatment dosing. Future studies should focus on safety, scalability, and long-term outcomes. Personalized medicine and collaborative research are essential. These developments may advance regenerative medicine and patient care.

This review highlights logical insights from current literature. The findings assist clinicians in selecting immunomodulatory strategies. Researchers may identify new targets for immune compatibility. Limitations include the scarcity of large-scale human trials and variability in experimental models.

## Data Availability

Not applicable.
